# Low-Cost HIV-1 Diagnosis and Quantification in Dried Blood Spots by Real Time PCR

**DOI:** 10.1371/journal.pone.0005819

**Published:** 2009-06-05

**Authors:** Nishaki Mehta, Sonia Trzmielina, Bareng A. S. Nonyane, Melissa N. Eliot, Rongheng Lin, Andrea S. Foulkes, Kristina McNeal, Arthur Ammann, Vindu Eulalievyolo, John L. Sullivan, Katherine Luzuriaga, Mohan Somasundaran

**Affiliations:** 1 Department of Pediatrics and Program in Molecular Medicine, University of Massachusetts Medical School, Worcester, Massachusetts, United States of America; 2 School of Public Health and Health Sciences, University of Massachusetts at Amherst, Amherst, Massachusetts, United States of America; 3 Global Strategies for HIV Prevention, San Rafael, California, United States of America; 4 Heal Africa Hospital, Goma, Congo; Singapore Immunology Network, Singapore

## Abstract

**Background:**

Rapid and cost-effective methods for HIV-1 diagnosis and viral load monitoring would greatly enhance the clinical management of HIV-1 infected adults and children in limited-resource settings. Recent recommendations to treat perinatally infected infants within the first year of life are feasible only if early diagnosis is routinely available. Dried blood spots (DBS) on filter paper are an easy and convenient way to collect and transport blood samples. A rapid and cost effective method to diagnose and quantify HIV-1 from DBS is urgently needed to facilitate early diagnosis of HIV-1 infection and monitoring of antiretroviral therapy.

**Methods and Findings:**

We have developed a real-time LightCycler (rtLC) PCR assay to detect and quantify HIV-1 from DBS. HIV-1 RNA extracted from DBS was amplified in a one-step, single-tube system using primers specific for long-terminal repeat sequences that are conserved across all HIV-1 clades. SYBR Green dye was used to quantify PCR amplicons and HIV-1 RNA copy numbers were determined from a standard curve generated using serially diluted known copies of HIV-1 RNA. This assay detected samples across clades, has a dynamic range of 5 log_10_, and %CV <8% up to 4 log_10_ dilution. Plasma HIV-1 RNA copy numbers obtained using this method correlated well with the Roche Ultrasensitive (r = 0.91) and branched DNA (r = 0.89) assays. The lower limit of detection (95%) was estimated to be 136 copies. The rtLC DBS assay was 2.5 fold rapid as well as 40-fold cheaper when compared to commercial assays. Adaptation of the assay into other real-time systems demonstrated similar performance.

**Conclusions:**

The accuracy, reliability, genotype inclusivity and affordability, along with the small volumes of blood required for the assay suggest that the rtLC DBS assay will be useful for early diagnosis and monitoring of pediatric HIV-1 infection in resource-limited settings.

## Introduction

It is estimated that 33.2 million people were infected with HIV-1 at the end of 2007; 2.5 million were children under 15 years of age, the majority of whom acquired infection through mother-to-child transmission (MTCT; [1]). Antiretroviral therapy (ART) is effective at blocking MTCT and can markedly improve the outcome of pediatric HIV-1 infection. However, efforts to provide widespread access to ART have been hampered by the limited availability of infant diagnostic methods.[2]–[6] Methods to diagnose and monitor HIV-1 infection in resource-poor settings are usually limited to serologic assays and CD4/CD8 counts.[2], [7], [8] However, antibody based assays can reliably guide diagnosis and management only after 18 months of age following clearance of passively transferred maternal antibodies.[9] PCR based nucleic acid amplification and quantification of HIV-1 is the gold standard for early HIV-1 diagnosis in infants and for evaluating ART efficacy.[2], [5], [10]–[16] Several commercial nucleic acid based molecular tests are available for HIV-1 diagnosis and viral load quantification.[15] These commercial assays require relatively large volumes of blood that need to be processed for plasma, stored, and transported under special conditions. The cost, run-time, training, maintenance and infrastructure needed to perform these assays have also limited their use in low resource settings.[6], [17], [18] Practical and reliable methods to obtain, store, and transport blood samples are necessary to develop cost effective early diagnostic assays in limited-resource settings. Spotting of whole blood onto filter paper offers technical and economic advantages over conventional venipuncture methods since it simplifies sample collection and transport to reference laboratories for diagnostic testing and viral load quantification.[17], [19]–[24]

In the present study, we describe a LightCycler-based real time PCR assay (rtLC) to quantify viral loads using RNA extracted from filter paper containing dried blood spots. DBS prepared either under controlled conditions in a laboratory setting by research technicians or in a clinical setting by health-professionals were successfully evaluated. This assay has comparable reproducibility, diagnostic accuracy, specificity and broader linear range at a lower cost compared to probe-based commercial assays. The rtLC HIV-1 DBS assay is also capable of detecting and quantifying different clades of HIV-1 without sacrificing sensitivity.

## Methods

### Patients and Reference Samples

#### Study Participants

In this prospective study, blood samples were drawn by trained healthcare professionals from 33 HIV-1 positive children who presented to the UMass Mother-Child HIV Program clinic for routine care between May 2005 and September 2008. Study participants ranged in age from 5 to 21 years. Four HIV-1 positive adults who were enrolled in a study of viral kinetics between 1999 and 2002 were also included in the study. Thirty patients were of U.S. origin and were infected with clade B HIV-1, while 7 were of non-U.S. origin, infected with non-B. Nineteen (51%) of the 37 HIV-1 patients were on ART and eight (22%) had undetectable RNA by Versant HIV-1 RNA 3.0 branched DNA (bDNA) assay (Siemens Healthcare Diagnostics) or Ultrasensitive Amplicor HIV-1 Monitor v1.5 assay (Roche Diagnostics) (Table 1). These tests were performed by trained technicians. Blood samples were also drawn from 44 HIV-1 negative individuals (27 adults, 17 children). Individual patient consent was obtained from study participants and guardians and no adverse events were associated with drawing of blood for the study. The Human Subjects Committee of the University of Massachusetts Medical School approved all studies.

**Table 1:** Characteristics of the HIV-1 infected patients of US and Non-US origin studied.

				Viral load (copies/ml)		
Patient ID	Country of origin/clade	Age of patient (yrs)	Patient on ART at visit	bDNA	Roche US	rtLC DBS
P-1014	US	17	No	8068	11024	16144
P-1008	US	21	No	45484	142176	52920
P-1023	US	16	No	68654	170000	90680
P-1039	US	12	No	2076	7001	5424
P-1025	US	17	No	<75	271	<37
P-1017	US	17	Yes	2910	7755	11208
P-1036	US	12	Yes	3144	6846	13120
P-1325	US	13	Yes	34332	92001	52760
P-1047	US	12	Yes	N/A	<50	<37
P-1020	US	18	Yes	<75	<50	<37
P-1031	US	14	Yes	N/A	20952	9960
P-1012	US	20	Yes	485	1323	5624
P-1048	US	10	Yes	N/A	<50	<37
P-1115	US	15	Yes	9843	17613	3228
P-1033	US	15	No	19579	20082	42580
P-1040	US	13	Yes	<75	<50	<37
P-1336	US	19	No	29908	39466	21468
P-1341	US	17	Yes	226	<50	<37
P-1046	US	12	Yes	739	589	4606
P-1319	US	8	Yes	11531	9853	8792
P-1117	US	12	Yes	N/A	8380	7129
P-1363	US	14	Yes	N/A	19900	6000
P-1107	US	19	NO	4940	11832	1771
P-1366	US	9	No	N/A	43617	36242
P-1044	US	13	Yes	2421	3396	15848
P-1339	US	7	Yes	298	<50	<37
A1	US/B	40	No	N/A	160070	214400
A2	US/B	52	No	N/A	898462	2379600
A3	US/B	48	No	N/A	136048	57140
A4	US/B	47	No	N/A	484018	786600
P-1347	Burundi/A	15	No	N/A	13605	26250
P-1211	Cambodia/AE	9	Yes	<75	<50	<37
MDOT 25	Cambodia/AE	8	N/A	N/A	220456	29480
MDOT 22	Cambodia/AE	1	N/A	N/A	271744	159120
P-1210	Liberia/AG	10	Yes	N/A	135417	166360
P-1359	Liberia/AG	13	No	12509	207236	122600
P-1334	Zambia/C	14	No	N/A	196285	26440

N/A = Not Available

ND = Not Detected

In addition, DBS samples were collected between February 2008 and January 2009 by heel-stick from 19 infants born to HIV-1 positive mothers at the HEAL Africa Hospital (Goma, Democratic Republic of Congo). Informed consent for testing was obtained from the infants' guardians. These studies were exempt under the guidelines of the Human Subjects Committee of the University of Massachusetts Medical School since the samples were received in the lab as coded, de-identified DBS with no traceable patient information.

#### Preparation of DBS

At the UMass clinic, whole blood was drawn by venipuncture and collected in tubes with EDTA. DBS were prepared by spotting 50 µl of whole blood onto filter paper (Whatman no. 903; formerly SS 903, Schleicher & Schuell, Keene, NH). For 6 of the 23 patients, cryopreserved plasma was spiked into donor HIV-1 negative blood to prepare DBS since whole blood was not available. The spotted filter papers were allowed to dry for at least 4 hours at room temperature in a hood and placed in individual ziplock bags containing a silica desiccant (Whatman, Schleicher & Schuell, Keene, NH). DBS were stored at −20°C until further processing and testing. The plasma was recovered from the remaining blood sample by centrifugation and stored at −80°C for quantification of viral load using commercial (Roche) RNA assays.

At the HEAL Africa Hospital (Goma, Democratic Republic of Congo) site, DBS samples collected by heel stick from infants born to HIV-1 positive mothers were stored and shipped to the laboratory at ambient temperature; upon receipt in the laboratory, the DBS samples were stored at −20°C until ready to be analyzed.

To better investigate whether the rtLC DBS assay could detect and quantify across clades, in addition to the Congo DBS specimens, we prepared dried spots of whole blood or plasma from 7 non-US origin patient samples (clades A, C, D, AE, AG) spiked into HIV-1 negative donor blood.

#### Genotype Inclusivity Studies

Genotypic clade determination of patient viruses was done using RT-PCR followed by nested amplification of were performed to amplify *env* and/or *gag* genes from plasma HIV-1 RNA. Amplified products were cloned for bi-directional DNA cycle sequencing using an ABI model automated sequencer. Phylogenetic analyses based on *env* and *gag* nucleotide sequences were used to determine the clade specificity.

#### DBS of International Panel of HIV-1 Isolates Representing Globally Prevalent Strains

Reference viruses (13 CCR5-tropic and 1 CXCR4-tropic) were obtained from the NIH AIDS Research and Reference Reagent Program, Division of AIDS, NIAID, NIH (Catalog #11412).[25] This panel represented 6 major globally prevalent HIV-1 clades (A, B, C, D, and circulating recombinant forms CRF01_AE and CRF02_AG).

RNA from these reference viral isolates was extracted using the QIAamp Viral RNA mini kit, according to manufacturer's instructions (Qiagen, Valencia, CA), eluted in 60 µl of elution buffer and then diluted 1∶1000. A DBS panel was prepared by mixing 60 µl of each virus isolate with 240 µl of seronegative donor whole blood and then spotting 50 µl of this spiked donor blood onto 903 filter paper. Using the protocol described below, this dried blood spot panel was extracted and run in the rtLC DBS assay.

#### Effect of Storage Time and Temperature on Stability of DBS Samples

Plasma samples of 12 patients from 5 countries with known viral loads were selected. Plasma (25 ul) was spiked into 25 ul of HIV-1 negative donor whole blood to prepare DBS. DBS at −20°C and 37°C storage temperatures were extracted on day 1 and day 7 post-preparation. Due to limited quantity of plasma, 8 out of 12 patient-DBS stored at these 2 temperatures were extracted only on day 7 post-preparation.

#### Preparation of Standard Curve

To ensure uniformity and reproducibility in the DBS preparation and extraction process, we prepared customized DBS standards with known viral copies. An HIV-1 clade B infected patient isolate with known viral load was selected and spiked into HIV negative donor whole blood to prepare DBS in five fold serial dilutions. A series of 5 independent extractions were performed and quantified using the rtLC DBS assay in a total of 25 independent runs.

#### RNA Extraction

Each DBS containing 50 µl of whole blood was cut into 4 equal pieces and incubated for 5 minutes in Tris-EDTA buffer (1.0 M Tris-HCl, 0.1 M EDTA) at room temperature. HIV-1 RNA was extracted from the filter paper using Trizol reagent as lysis solution according to the manufacturer's instructions (Invitrogen, Carlsbad, CA). Glycogen (200 µg) was added to the lysis reagent as a carrier to facilitate RNA precipitation for each DBS extraction. After removing the residual filter paper, 1-bromo-2-chloropropane (BCP) was used to separate the extracted RNA from the organic phase. RNA was ethanol precipitated, eluted and further reconstituted in 40 µl of Rnase-free water containing 40 units of Rnasin Plus, an Rnase inhibitor (Promega, Madison, WI).

#### DNA Extraction

One spot each of DBS filter paper was processed to extract DNA using the previously described resin-based Chelex (Bio-Rad, Hercules, CA) method.[26] Briefly, the paper punched from one entire DBS was immersed in 1 mL of whole blood specimen wash solution (Amplicor HIV-1 Monitor test v1.5, Roche Molecular Systems) and rocked for 2 hours at room temperature. After a quick spin for 5 minutes at high speed, the red-tinged buffer was removed and discarded. The DBS paper was washed once, and then immersed in 250ul of Chelex resin resuspended in DNA dilution buffer (10% v/v in 10 mM Tris buffer, pH 8.3 containing 50 mM KCl). DNA kit Internal Control (3.3ul) was added to the DBS sample, mixed frequently to keep the resin in suspension, and was heated to 100°C for 1 hour, with a 10 second vortex after the first 30 minutes of heating. After a spin for 3 minutes to pellet the resin, the supernatant containing the extracted DNA was removed and stored at −80°C until HIV-reactivity of the extract was determined using Roche Amplicor HIV-1 DNA Amplicor 1.5 v kit according to a previously published protocol.[26] The cellular equivalents of DNA in each test sample were determined in a real-time Taqman PCR assay by probing for CCR5 copy number using forward primer 5′-GCTGTGTTTGCGTCTCTCCCAGGA-3′ and reverse primer 5′-CTCACAGCCCTGTGCCTCTTCTTC-3′, and the corresponding fluorogenic probe 5′FAM-AGCAGCGGCAGGACCAGCCCCAAG-TAMRA 3′. Known copies of plasmid carrying the CCR5 gene were used to generate the standard curve, from which the number of CCR5 copies and cellular equivalents were determined for each sample. Real-time PCR analyses on RNA extracted from infant DBS was performed as described below.

#### Real-time PCR

Real-time PCR amplification of HIV-1 RNA was performed in a one-step, single-tube closed system of 32 sample format using the LC-32 Roche LightCycler (Roche, Indianapolis, IN). All the samples were tested in duplicate in a 20 µl total reaction volume containing 16 µl of PCR reaction mix (Quantitect SYBR Green RT-PCR kit [Qiagen, Valencia, CA]; 0.5 µM each of forward and reverse oligonucleotide primer pairs) and 4 µl of the template. The primers were specific to a conserved region of HIV-1 LTR: 5′-GRAACCCACTGCTTAASSCTCAA-3′ (LTR sense; position 506 of HxB2) and 5′-TGTTCGGGCGCCACTGCTAGAGA-3′ (LTR antisense; position 626 of HxB2).[27] The PCR reaction was performed according to the following cycling parameters: 1) Reverse-Transcription: 50°C 20 minutes, ramp 20°C/second; 2) Activation: 95°C 15 minutes, ramp 20°C/second; 3) Amplification: 50 cycles a) 94°C 10 seconds, ramp 20°C/second, b) 52°C 20 seconds, ramp 20°C/second, and c) 72°C 20 second, ramp 2°C/second (single data collection); 4) Melting: a) 92°C 0 second, ramp 20°C/second, b) 57°C 15 seconds, ramp 20°C/second, and c) 92°C 0 second, ramp 0.1°C/second (continuous data collection); 5) Cooling: 40°C 30 seconds, ramp 20°C/second.

HIV-1 specific amplicons were detected using SYBR Green technology according to manufacturer's instructions (Qiagen, Valencia, CA). The number of HIV-1 RNA copies in each test template was measured by its threshold cycle (C_t_) and determined from the standard curve of serially diluted customized DBS standards using software for data analysis (Sequence Detector version 1.6, PE Applied Biosystems). For each experiment, a standard curve was generated from serial endpoint dilutions (586,000 to 37 copies) of the customized DBS standards. The threshold cycle values were plotted against copy numbers to construct the standard curve. Quantification of HIV-1 RNA in each test sample was back calculated and viral load was expressed as copies/ml. Using the protocol described above, DBS was extracted, run and analyzed in the rtLC DBS assay by personnel with molecular and virology expertise who were blind to the results.

Rnase-free water (4 µl) was routinely added instead of test sample to 16 µl of the master mix and used as a no-template control for every run. At the end of the assay, the specificity of each amplified product was ascertained by means of melting curve analysis. This eliminated false positive detections due to primer dimers or non-specific amplicons. To confirm that there was no DNA contamination in the input RNA and to assess the specificity of the reverse transcribed rtLC DBS products, initial assays included PCR reactions without the addition of reverse-transcriptase enzyme. Gel electrophoresis of the amplicons was initially performed to further confirm the specificity of the products. Each sample was tested in replicates and a second DBS was independently extracted and run to verify a positive result. The test was repeated if the sample had indeterminate results, and if necessary a new spot was extracted and tested if the results of the repeat test were also indeterminate. Quality control for each experiment was assessed by the performances of the standard curve and the negative control. All DBS prepared by trained personnel were tested for this study as long as multiple filter spots were available for each patient, and the blood spots were good quality with no hemolysis or clots.

To determine whether viral load data using the Roche LightCycler system were comparable to viral load data obtained by using MyiQ™ Single-Color Real-Time I-Cycler PCR Detection System, (Bio-Rad, Hercules, CA), cDNA synthesis was carried out with the iScript cDNA Synthesis Kit (Bio-Rad, Hercules, CA). Select patient and standard DBS-RNA were used to synthesize cDNA, and then amplified and quantified using the iQ SYBR Green Supermix Kit (Bio-Rad, Hercules, CA) by using these parameters: 3 min @95C, 50 cycles of 10 sec @94C, 20 sec @52C, 20 sec @72C, and final extension for 20 sec @72C. Results were analyzed using MyiQ software, version 1.0.410 (Bio-Rad, Hercules, CA).

#### Sensitivity and Linearity of the Assay

Linearity of the assay was evaluated using serial dilutions of the customized standard DBS described earlier (586,000 copies to 37 copies).

#### Statistical Analyses

The customized DBS standards serial dilutions were tested in 25 separate runs to determine the threshold, inter-assay precision and linearity of the rtLC DBS assay. Trained statisticians performed data analyses. Probit analysis was used to determine 95% and 99% detection limits. The likelihood ratio test was used to evaluate the effect of extractions and runs; the fitness was measured by R-squared value. Spearman correlation coefficients were calculated to determine the relationship between the HIV-1 RNA levels quantified by the Roche Ultrasensitive and bDNA assays with the rtLC DBS technique. The sensitivity of the rtLC DBS assay was calculated as the number of positive results divided by the total number of samples from infected patients who had plasma viral load above the threshold level of detection of commercial assays (bDNA and Roche Ultrasensitive) expressed as a percentage. The specificity was calculated as the number of negative samples divided by the total number of known negative specimens obtained from normal healthy donors and uninfected infants expressed as a percentage. Paired Wilcoxon tests were used to determine the effect of temperature and time on RNA stability and assay performance.

## Results

### Linear Range, Detection Limits and Inter-assay Precision of the rtLC DBS Assay

**Figure 1:**
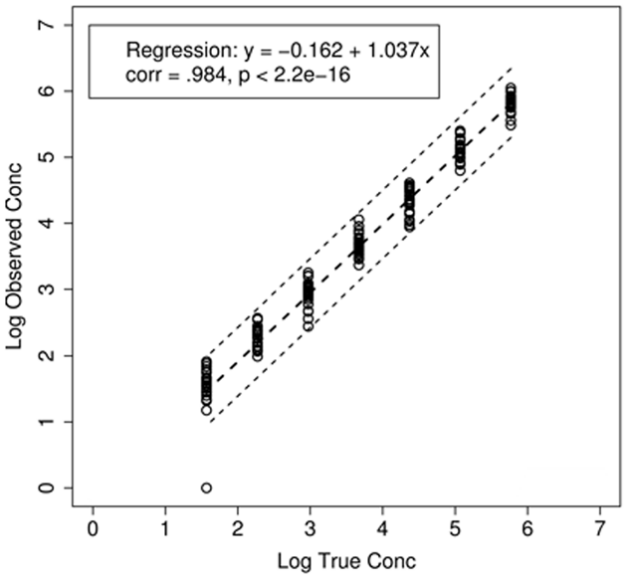
Linearity of the rtLC DBS assay using dilutions of known concentrations of HIV-1 from a clade B infected patient. The concentrations quantified by rtLC DBS assay obtained from 25 experiments were plotted against true standard plasmid concentrations. They ranged from 37 copies to 586,000 copies/reaction sample with a correlation of 0.984 (*P*<0.001).

The linear dynamic range of the rtLC DBS assay was initially assessed using a 5 log_10_ dilution series of the customized standard DBS. The assay was shown to be linear over the entire range of 586,000 to 37 copies. A linear regression of the rtLC DBS customized standards copies on true concentrations yielded a correlation coefficient of 0.984 (*P*<0.001) and the fitted model is shown in Figure 1. The slope of 1.037 closely approximates the theoretical maximum amplification efficiency of 100%; the fitted slope is slightly greater than 1 due to the several undetected cases in low range. To estimate the detection limit of the assay, we performed 22 extra runs at low concentrations (7, 17, 37, 83, 187 copies), which spread evenly in log scale and contained two concentration 37 and 187 that were already in design. We used all available data at concentrations from 7 to 937 to fit the probit model to get estimations for 95% detection limit as 135, with 95% confidence interval [82, 223]; 99% detection limit was estimated as 292, with 95% confidence interval [147, 583] (Table 2).

**Table 2:** Calculation of the 95% and 99% detection limit of the rtLC DBS assay using probit analysis.

Actual concentration (copies/reaction)	Measured concentration (median copies/reaction)	Number of positive reactions/total test reactions (%)
Four concentrations > = 4688	--	100/100(100%)
**292**	**248**	**99%** *
937	941	25/25 (100%)
187	188	44/46 (95.7%)
**136**	**112**	**95%** *
83	56	17/22 (77.3%)
37	39	38/47 (80.9%)
17	9	11/22 (50%)
7	0	2/22 (9%)

* The assay sensitivity was calculated using 22 replicate measurements for low range concentrations between 7 and 187 copies. Results from concentration 37, 187 and 937 copies from 25 runs of standard curve data were also used for Probit fitting. The data used for the analyses are shown in regular typeface and the estimated 99% and 95% detection limits are shown in **boldface**.

To evaluate the inter-assay precision of the assay, the data on the customized DBS standards of known concentrations (586,000 to 37 copies) were analyzed across 25 independent experiments. The results of the statistical analysis for the standard deviation (SD) and percent coefficient of variation (%CV) at each concentration level are shown in Table 3. The coefficient of variation increased dramatically when the true concentration was approximately equal to or below the assay's detection limit.

**Table 3:** Precision of the rtLC DBS assay: The standard deviation and the percent coefficient of variation were calculated using data from 25 independent experiments.

Actual concentration (log copies/reaction)	Measured concentration (average, log copies/reaction)	Standard deviation (SD)	% Coefficient of variation (% CV)
5.77	5.79	0.14	2.5
5.07	5.15	0.15	3.0
4.37	4.28	0.18	4.2
3.67	3.69	0.15	4.0
2.97	2.85	0.49	17.1
2.27	2.20	0.22	10.1
1.57	1.43	0.55	38.7

### Sensitivity and Specificity of the rtLC DBS Assay

The clinical sensitivity of the assay was determined using DBS specimens of 32 patients with known infection and viral loads above the detection threshold of the bDNA and Roche Ultrasensitive assays (Table 1). All 32 samples were positive using the rtLC DBS assay, yielding a clinical sensitivity of 100% for this cohort. An additional 5 DBS samples drawn from infants of indeterminate HIV-1 infection status tested positive on the rtLC DBS assay; all 5 were also positive on the commercial DNA assay. The clinical specificity of the rtLC DBS assay was determined using DBS specimens from 27 healthy HIV-1 negative adult donors and 17 HIV-1 negative infants born to HIV-1 positive women. All of these samples were negative for HIV-1 RNA, resulting in a clinical specificity of 100%. The information on the clinical specificity of rtLC DBS assay in HIV negative donors is provided in Table S1 in the supplementary section.

### rtLC Assays Using Samples Collected in the Field

DBS samples from infants born to HIV positive women were also prepared at a clinical site in Goma, Congo. The range of cell equivalents across these Congo DBS samples was 2,769 to 201,116 cells/DBS (median 114,980 cells/DBS) when CCR5 copies were determined in the DNA extracts as shown in Table 4. An aliquot of RNA for each sample was spiked into normal human plasma and run on a Roche Amplicor HIV-1 Monitor Ultrasensitive assay. DNA testing determined that 5 of 19 samples were positive for proviral DNA. These same 5 samples were positive using the rtLC DBS assay (range = 11,084 to 1,123,400; median 196,200 copies/ml) with RNA copy numbers similar to the values obtained on the Roche Amplicor assay.

**Table 4:** Results of the infant DBS samples prepared onsite at HEAL Africa Hospital in Goma, Democratic Republic of Congo.

			Viral load (copies/ml)	
Congo ID	Cells/DBS	HIV-1 provirus	Roche US	rtLC DBS
CO323D	20711	Neg	NT	Neg
CO473D	63879	Neg	NT	Neg
CO472D	44936	Neg	Neg	Neg
CO259D	87188	Neg	NT	Neg
CO319	196893	Neg	NT	Neg
CO403	152982	Neg	neg	Neg
CO404	126770	Pos	2240	11084
CO413	114980	Pos	436160	196200
CO460D	86618	Neg	neg	Neg
CO487D	201116	Pos	82560	179520
CO534	161767	Neg	NT	Neg
CO219D	2769	Neg	NT	Neg
CO461D	178125	Pos	1467520	1,123,400
CO533D	94875	Neg	Neg	Neg
CO513D	17741	Neg	NT	Neg
CO530D	13388	Neg	NT	Neg
CO599D	95625	Pos	617600	206,460
CO644D	39048	Neg	NT	Neg
CO647D	32887	Neg	NT	Neg

Pos, positive; Neg, negative; NT, not tested

### Genotype Inclusivity

The rtLC DBS assay successfully detected HIV-1 RNA in each of the DBS samples prepared from 14 viral isolates representing an international HIV-1 reference panel (clades A, B, C, D and CRF-AE and CRF-AG).

The rtLC DBS assay also successfully detected and quantified HIV-1 RNA in DBS samples prepared in our lab using whole blood samples from 23 patients (US origin) infected with clade B virus (Table 1), and 6 patients (non-US origin) infected with non-clade B virus (Table 1). Finally, the rtLC DBS assay successfully detected HIV-1 nucleic acids in the blood of all 5 HIV-1 positive Congo infant DBS samples that were prepared in the field and shipped at ambient temperature (Table 4).

### Comparison of Viral Load Measurements Using the rtLC DBS Assay and Commercial Viral Load Assays

Plasma viral RNA copy numbers determined by the rtLC DBS assay were compared to results obtained using commercial assays (bDNA and Amplicor). The Spearman coefficients of rtLC DBS with the Roche and bDNA assays were 0.91 and 0.89 respectively.

In a pilot study using the i-Cylcer real-time PCR system to quantify viral load from select patient and standard DBS RNA preparations, and using Sybr Green dye for detection, we demonstrated comparable performance to that of the LightCycler system (Figure S1; Supplementary section). A significant correlation between i-Cycler and LightCycler based viral loads was observed in DBS specimens (Spearman Ranks correlation, p<0.0001) suggesting that our LightCycler-based DBS assay will have universal applicability.

### Effect of Ambient Temperature on Stability of HIV-1 RNA

**Figure 2:**
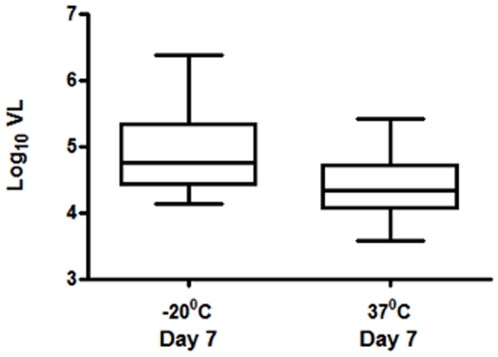
Comparison of viral load from DBS of 12 patients, stored at −20°C and 37°C, extracted on day 7 post-preparation and quantified by rtLC DBS assay.

To evaluate the performance of this assay in limited resource settings where storage and shipment of DBS at ambient/room-temperature (>25°C) is the norm, we investigated the effect of 37°C temperature on the stability of DBS/RNA, by comparing the detection of HIV-1 RNA in DBS samples stored at −20°C or 37°C. We also evaluated 7 days of storage at 37°C to emulate shipment of DBS from the point of preparation (clinic) to a tertiary referral center (reference-laboratory), and compared the assay results to those of identical DBS stored promptly at −20°C. No statistically significant difference in viral load was observed for 12 samples stored either at -20°C or 37°C (Wilcoxon signed rank test, p = 0.06) (Figure 2). Further, to evaluate for potential loss of RNA over time in DBS samples stored at −20°C and 37°C, viral load was determined on RNA samples extracted day 1 and day 7 post-preparation of DBS. The data (available for 8 patients) for days 1 and 7 were comparable irrespective of the storage temperature (paired Wilcoxon test; data not shown).

### Extraction and Inter-assay Efficiency

The fitness measured by R-squared value in the simplest model, which includes only the true concentration as predictor, is 0.955, and R-squared values from models that include extraction and run effects were 0.9959 (extraction and run) and 0.9948 (extraction), respectively. The improvement of fitness by including extra extraction specific parameters and run specific parameters are marginal, and we conclude that true concentration explains most (95.5% percent) of the variation in observed concentration, and the influence of extractions and runs are limited.

### Measurement of Plasma HIV-1 RNA over time in Patients on ART

**Figure 3:**
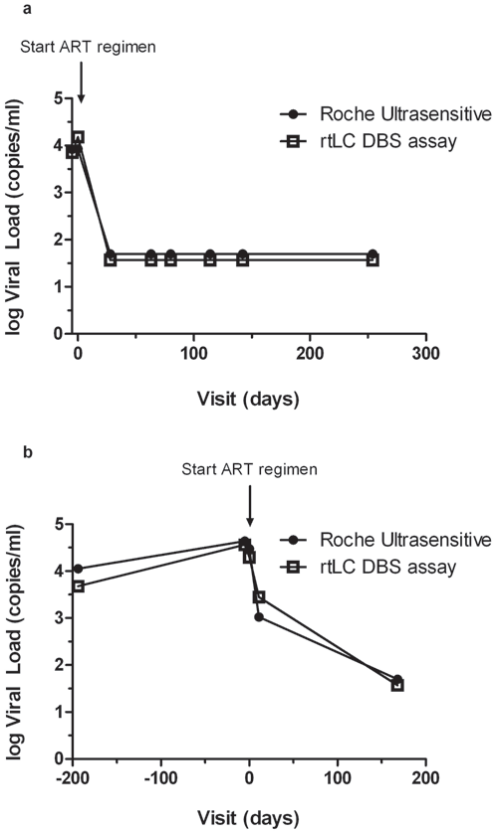
a & b. Comparison of viral load measurements quantified by rtLC DBS and Roche Ultrasensitive assays for patients on therapy followed longitudinally. (Fig 3a – Patient 1117; Fig 3b – Patient 1366).

The rtLC DBS assay was used to monitor plasma HIV-1 RNA levels in two patients for up to one year on ART (Figures 3a and 3b). The rtLC DBS assay showed good correlation with the Roche Ultrasensitive assay for the longitudinal follow-up of these two patients, suggesting that it may also be useful for monitoring viral load in patients on ART.

## Discussion

In this study, we used a nucleic acid based real-time PCR assay to detect and quantify plasma HIV-1 copy numbers on samples from 56 HIV-1 infected patients utilizing the DBS format. The assay results were comparable and correlated well with commercially available viral load assays (Siemens bDNA, Spearman *r* = 0.89 and Roche Ultrasensitive Amplicor, Spearman *r* = 0.91). In the patient cohort analyzed, the assay successfully detected all positive samples. The calculated specificity using known negative samples was 100%. The estimated 95% detection threshold was 136 copies and the dynamic range of the assay was 5 log_10_. Finally, the assay successfully detected four major subtypes and 2 CRF of HIV-1.

A little over a decade ago, we demonstrated the utility of early combination antiretroviral therapy (ARV) in infants.[28], [29] HIV infection is associated with particularly high morbidity and mortality in limited-resource settings and a recent randomized trial conducted in South Africa demonstrated reduced morbidity and mortality in infants treated with early combination ARV.[30] In a recent WHO consultants' meeting, revision of current guidelines was recommended to include routine early diagnosis and treatment of HIV positive infants under 1 year of age.[31] Nucleic acid based testing is the gold standard for early diagnosis. Commercially available assays (bDNA, Roche Amplicor Ultrasensitive, and Cobas) are relatively expensive and require significant infrastructure and technical expertise to allow transfer of technology to resource-limited settings.[2], [8] Hence access to nucleic acid testing in these settings is currently very limited.[6], [17], [18], [32], [33]

Studies to detect and quantify HIV-1 have traditionally involved two-step, nested or probe based PCR [34]–[37]; more recently, real time PCR has been used for HIV detection and quantification.[6], [22], [24], [34], [38], [39] Multiple investigators have documented the utility of DBS sample collection for early HIV-1 detection in infants, viral load monitoring, and surveillance of seropositivity and drug resistance in laboratories and clinics that lack facilities for refrigeration or sample processing.[8], [17], [20], [22]–[24], [38], [40]–[52] The DBS format greatly facilitates the logistics of sample collection, processing, and shipping for limited resource settings. Whole blood saved as DBS can be transported or mailed to reference laboratories without refrigeration and has low biohazard risk. Optimal storage conditions for DBS and long term stability of DNA and RNA from DBS under different storage conditions have been extensively documented.[23], [44], [47], [51], [53] Our pilot studies with a small cohort of patient samples to assess the effect of temperature and time, albeit one week, on the stability of RNA demonstrated no difference, and were in agreement with previous larger studies.[39], [54]–[56] Comparable efficacy using multiple extractions and runs of our customized standards support the reliability of the DBS format and the rtLC DBS assay.

The rtLC DBS assay described herein is a one-step, walk away technique. Automation of this assay provides potential for high throughput with very small sample volumes, which makes the assay suitable for use in infants and children from whom one often has access to only small blood volumes. It is an assay system which will be cost-effective and easily adaptable to limited-resource settings, where the majority of new HIV-1 infections are seen today. The detection of PCR products by SYBR Green ensures good sensitivity. SYBR Green is relatively inexpensive compared to probe-based detection, and in general, SYBR Green detection is one cycle threshold or so more sensitive than probe-based assays.

Several previous studies have utilized SYBR Green for HIV-1 diagnosis and are reviewed by Espy et al.[57] The efficiency of this dye-based assay is also comparable with the currently available diagnostic assays on HIV. Aside from use in HIV diagnosis and quantification, SYBR Green has been widely used to detect and quantify diverse human pathogens.[58]–[65] The data in these reports strongly support the utility and reliability of SYBR Green for detection of specific PCR amplicons above the background. The use of degenerate LTR primers in the rtLC DBS assay allows for a wide range of genotype inclusivity.

Compared to commercial assays, the rtLC DBS assay is rapid and cost-effective. The equipment used in the rtLC assay is self-contained, occupies minimal bench-space and doesn't require accessory equipments (such as a plate washer, optical density reader, and incubator; Table 5). Aside from the initial cost of obtaining the LightCycler instrument, a comparative analysis of assay costs and technician time reveals a 40-fold decrease in cost as well as 2.5 fold decrease in technician time (4.5 hours) associated with the rtLC DBS when compared to commercial kit based PCR assays (Table 6).

**Table 5:** Infrastructure requirements of commercial versus rtLC DBS assays for viral load determination.

Infrastructure	Commercial assay	rtLC DBS assay
Bio-safety cabinet	1	1
Extraction Hood	1	1
Refrigerated Centrifuge	1	1
PCR Hood	2	1
Thermocycler (LightCycler)	1	1
Freezer Storage (in Celsius)	−70	−20
Lab space (dedicated areas)	5	3
Plate washer	Yes	No
Incubator	Yes	No
Plate Optical Density reader	Yes	No

**Table 6:** Cost and time comparisons of commercial verses rtLC DBS assays for viral load determination.

Factors considered	Commercial assay	rtLC DBS assay	Fold decrease
Cost/sample ($)	200	4.55	40
Tech time (hrs)	7.5	3	2.5
Blood volume (ul)	3000	50	64

The reduced equipment requirements, personnel hours, and costs compared to the commercial ‘gold’ standard assays make the rtLC DBS assay attractive for transfer to and use in resource-limited settings. The success of the pilot field study on DBS from Congo emphasizes the utility and applicability of our assay although further studies with larger sample sizes are definitely warranted.

In summary, we have utilized a real-time LightCycler based PCR assay on small volumes of whole blood dried on filter paper to successfully detect and quantify viral loads across different HIV-1 clades. The use of dried blood spots provides a simple and inexpensive means for obtaining blood samples for analysis that minimizes the risk for contamination while maximizing the ability to obtain timely results. A major advantage of the rtLC DBS assay is that the amplification, real-time detection and quantification, and confirmation of amplicon-specificity by melting curve analysis are performed in a one-step, closed-tube format. In addition, viral load tests by rtLC DBS assay are substantially less expensive and logistically less intensive than commercial assays. Preliminary data suggest the adaptability of the assay into other real-time systems. Validation of these results with larger field studies would constitute a more robust evaluation and will have major implications for early diagnosis, disease management, and epidemiological- or resistance- surveillance studies in limited resource settings.

## Supporting Information

Table S1 (0.04 MB DOC)

Figure S1 Spearman Ranks correlation between i−Cycler (iC) and LightCycler (LC) based real−time PCR systems to quantify HIV 1 viral loads in 21 patients and 8 standard DBS (*p*<0.0001).
(0.04 MB TIF)
