# Supplementary material for: Low-Cost HIV-1 Diagnosis and Quantification in Dried Blood Spots by Real Time PCR
Source: PLoS One. 2009 Jun 5;4(6):e5819. doi: 10.1371/journal.pone.0005819 (PMC2688035; doi:10.1371/journal.pone.0005819)
Supplement: Table S1 — (0.04 MB DOC) [file pone.0005819.s001.doc]

| **TABLE S1. Clinical specificity of the rtLC DBS assay: Summary of HIV negative samples with undetectable viral loads.** | | |
| --- | --- | --- |
|  |  |  |
| **HIV-1 negative samples** | **Country of origin** | **Sample number** |
| Seronegative adult donors | U.S. | 27 |
| DNA negative children born to HIV infected mothers | U.S. | 8 |
|  | Congo | 9 |
|  | | |
| **Total:** | | **44** |
