# Supplementary figures and images for: Low-Cost HIV-1 Diagnosis and Quantification in Dried Blood Spots by Real Time PCR
Source: PLoS One. 2009 Jun 5;4(6):e5819. doi: 10.1371/journal.pone.0005819 (PMC2688035; doi:10.1371/journal.pone.0005819)

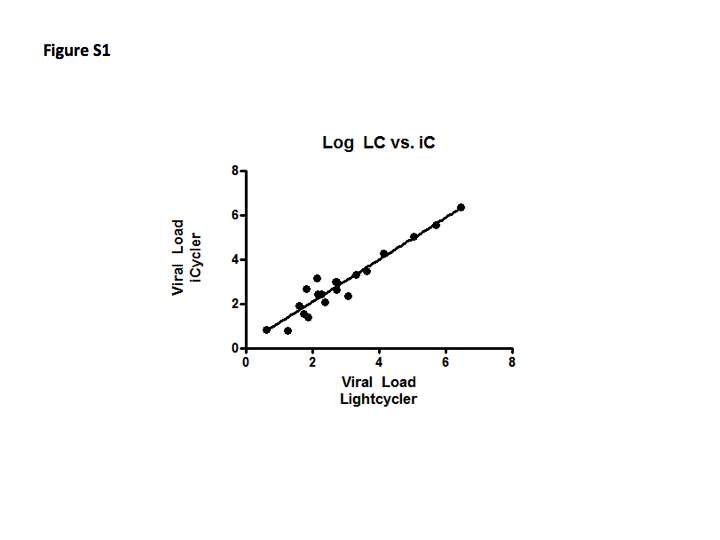

Supplement: Figure S1 — Spearman Ranks correlation between i−Cycler (iC) and LightCycler (LC) based real−time PCR systems to quantify HIV 1 viral loads in 21 patients and 8 standard DBS (p<0.0001). (0.04 MB TIF) [file pone.0005819.s002.tif]
